# Identification of high-risk COVID-19 patients using machine learning

**DOI:** 10.1371/journal.pone.0257234

**Published:** 2021-09-20

**Authors:** Mario A. Quiroz-Juárez, Armando Torres-Gómez, Irma Hoyo-Ulloa, Roberto de J. León-Montiel, Alfred B. U’Ren

**Affiliations:** 1 Departamento de Física, Universidad Autónoma Metropolitana Unidad Iztapalapa, Ciudad de México, México; 2 ABC Medical Center, Ciudad de México, México; 3 Instituto de Ciencias Nucleares, Universidad Nacional Autónoma de México, Ciudad de México, México; University of Pennsylvania, UNITED STATES

## Abstract

The current COVID-19 public health crisis, caused by SARS-CoV-2 (severe acute respiratory syndrome coronavirus 2), has produced a devastating toll both in terms of human life loss and economic disruption. In this paper we present a machine-learning algorithm capable of identifying whether a given patient (actually infected or suspected to be infected) is more likely to survive than to die, or vice-versa. We train this algorithm with historical data, including medical history, demographic data, as well as COVID-19-related information. This is extracted from a database of confirmed and suspected COVID-19 infections in Mexico, constituting the official COVID-19 data compiled and made publicly available by the Mexican Federal Government. We demonstrate that the proposed method can detect high-risk patients with high accuracy, in each of four identified clinical stages, thus improving hospital capacity planning and timely treatment. Furthermore, we show that our method can be extended to provide optimal estimators for hypothesis-testing techniques commonly-used in biological and medical statistics. We believe that our work could be of use in the context of the current pandemic in assisting medical professionals with real-time assessments so as to determine health care priorities.

## 1 Introduction

Coronavirus infectious disease (COVID-19) is a recently discovered illness caused by severe acute respiratory syndrome coronavirus 2 (SARS-CoV-2). As of the first week of February 2021, over 106 million SARS-CoV-2 infections and over 2.3 million deaths have been registered worldwide, in the worst pandemic to afflict humanity since the so-called Spanish flu of 1918, which has overwhelmed the world’s health care systems and caused severe economic disruption.

As a response to this international public health crisis, scientists and clinicians have made enormous efforts in the last few months to generate new knowledge and to develop technological tools that may help in combating this infectious disease and mitigate its effects. Some of these efforts include the development of drugs and vaccines [1–4], the construction of epidemiological models to forecast the dynamics of disease spreading in the population [5–8], the development of mobile-device applications for tracking infected patients and new cases [9–11], and the development of strategies and the application of new technologies to manage the resources and capacities in hospitals [12–14].

An emergency non-pharmaceutical prevention measure adopted in many countries has been the reduction or suspension of non-essential activities so as to reduce both the rate of new infections [15] and the risk of exceeding hospital capacities. Undoubtedly, the ability to rapidly identify high-risk patients and/or correctly assign health care priorities is critical, in the first case so as to improve hospital capacity planning and in the second case for providing timely treatment for patients [16]. In this regard, artificial intelligence methods have been recognized as a powerful and promising technology that can help not only in the identification of the fatality risk of a given patient seeking medical attention [17, 18], but also for the diagnosis process [19–22], prediction of disease spreading dynamics [23–27], and tracking of infected patients as well as likely future patients [28].

Machine learning is a branch of the artificial intelligence field which seeks to endow computers with a “learning capacity” using well-defined algorithms, to improve performance or make accurate predictions. Typically, these algorithms learn from past information available, introduced in the form of labeled training sets. The supervised learning algorithms use these labeled training sets to optimize the parameters of a statistical model so that a loss function is minimized. The trained model is then able to effectively make predictions using as input data which have never been used in the training phase. Of course, the quality and size of the data-sets used are crucial in ensuring the adequate performance of the algorithm [29, 30]. During the course of the current pandemic machine learning has been used to develop different algorithms that seek to identify, at an early stage, patients who are likely to become infected. These approaches make predictions relying on basic patient information, clinical symptoms [31–33], as well as travel history [34] and discharge time of hospitalized patients [16]. Souza *et al*. [31] have presented a study for the early identification of patients who can develop severe COVID-19 symptoms, using supervised machine learning algorithms such as logistic regression, linear discriminant analysis, naive Bayes, k-nearest neighbors, decision trees, XGBOOST, and support vector machine. The machine learning methods were trained using a publicly available database pertaining to Brazil, which includes individual basic information such as gender and age range, symptoms, comorbidities, and recent traveling history. The authors report that the disease outcome can be predicted with a ROC area under curve (AUC) of 0.92, a sensitivity of 0.88, and a specificity of 0.82. The study by Dan Assaf *et al*. [32] focuses on the identification of patients at risk for deterioration during their hospital stay using a database from a tertiary medical center. In their work, the authors train three different machine-learning methods (neural networks, random forest and classification, and regression tree) from historical and clinical variables such as APACHE II score, white blood cell count, time from symptoms to admission, oxygen saturation, and blood lymphocytes count. The results show 88.0% sensitivity, 92.7% specificity, and 92.0% accuracy. Li Yan *et al*. [33] propose a decision rule based on the supervised XGBoost classifier to predict patients at the highest risk. The predictive model is originally trained with three characteristics: lactic dehydrogenase (LDH), lymphocytes, and high-sensitivity C-reactive protein (hs-CRP). The results show that the model can accurately identify the outcome of patients with more than 90% accuracy. Some other efforts focus on identifying patients requiring specialized care, namely hospitalization and/or specialized care units [35–37], or patients at a higher fatality risk [38, 39]. For example, Bezzan and Rocco [35] use laboratory data, collected from Sirio Libanes Hospital in Brazil, to identify patients requiring special care at the hospital and to predict lengths of stay at the specialized care units. The authors test several ML algorithms to select the best performance. The final selection is the XGBOOST algorithm for both targets, which achieves 0.94 ROC AUC for the first target and 0.77 for the second target. Pourhomayoun and Shakibi [39] present machine learning algorithms, including support vector machines, neural networks, random forest, decision tree, logistic regression, and k-nearest neighbors, to predict the mortality rate of COVID-19 patients. To train the algorithms, the authors use laboratory-confirmed cases belonging to 76 countries around the world. The dataset used contains demographic data, travel history, general medical information such as comorbidities, and symptoms. Their results show that the neural network algorithm achieves the best performance with an accuracy of 93.75%.

In this work, we introduce a machine-learning algorithm which effectively identifies high-risk patients among those that may have been exposed to the SARS-CoV-2 virus. Our method employs a supervised artificial neural network which predicts whether a given patient belongs to one of two classes: *class 1*, which represents those patients who are more likely to survive than to die, and *class 2* which represents those patients who are more likely to die than to survive. In order to achieve this classification, we rely on a database with information about past infections (along with suspected infections), from which we extract a 28-element characteristics vector for each patient. The characteristics include information about comorbidities, patient demographic data, as well as recent COVID-19-related medical information. Importantly, although the database does not include information related to clinical manifestations, diagnosis, and laboratory test findings, our method can still detect high-risk COVID-19 patients successfully. In our algorithm, we apply our rapid identification of patients (belonging either to class 1 or class 2) at any of four clearly-defined clinical stages of the treatment process, ranging from stage 1 at which a patient first becomes ill and seeks medical attention, to stage 4 at which not only is the patient hospitalized but requires specialized attention. We have trained a neural network for each of the four clinical stages, with data corresponding to a characteristics subset, with later clinical stages having access to a larger fraction of the 28 characteristics as they become known during the treatment process. Our algorithm is able to classify patients with high accuracy at each of the four clinical stages, with the accuracy value increasing with the progression from one stage to the next. Remarkably, we demonstrate that our algorithm can provide an optimal estimator for hypothesis-testing techniques commonly-used in biological and medical statistics. This creates a bridge between machine-learning algorithms and clinical medicine that allows for the introduction of a series of novel strategies, with applications in different clinical scenarios, in a familiar language for clinicians. We believe that this technology can be a powerful tool for medical resource allocation and hospital capacity planning, by making correct, real-time assessments of mortality risks, given the highly specific characteristics of each particular patient.

## 2 Materials and methods

### 2.1 Data

Our studies presented in this paper are based on the publicly-available database of COVID-19 patients from the Mexican Federal Government. This database, which includes all officially reported confirmed and suspected COVID-19 cases reported in Mexico, is available in the ‘Statistical Morbidity Yearbooks’ (Anuarios Estadísticos de Morbilidad) published by the General Council of Epidemiology (Dirección General de Epidemiología), part of the Health Ministry (Secretaría de Salud), Mexican Federal Government [40]. The data in the reports are provided by the National Epidemiologic Surveillance System, which comprises 475 health monitoring units of viral respiratory diseases (USMERs) distributed across the country. So as to ensure the representativeness of the data sample, the USMERs are chosen by taking into account demographic and climatic factors that may cause variations in the transmission conditions. Thus, these units provide attention to representative populations from different age and socioeconomic groups. The data recording process varies from institution to institution according to local procedures and staff availability. For this reason, all submitted data are considered preliminary and are subject to review and validation by the Health Ministry. Amongst patients who seek medical attention and/or are tested for COVID-19, the database ends up including in principle all of those either with a confirmed infection or showing symptoms, indicating a possible infection. Each patient in the database is classified into seven groups: i) confirmed COVID-19 infection through a positive real-time reverse transcription-polymerase chain reaction (RT-PCR) test and/or a positive COVID-19 antigen test, ii) clinical-epidemiological association in the absence of a valid test result (i.e. patient reported contact with a confirmed COVID-19 patient), iii) for deceased patients without a valid test result, designation by a special committee, iv) negative RT-PCR test result and/or negative antigen test result, v) laboratory result with an invalid result, vi) unprocessed laboratory result, as well as vii) suspected COVID-19 infection with laboratory test in process [41, 42]. Importantly, so as to avoid duplicated patients, a unique identification number is assigned to each patient. Although the database is updated daily, there is a reporting lag close to two weeks. For the period from April 12^th^, 2020 to January 31^th^, 2021 this database contains a historical record of 4,700,464 patients who have received medical attention at both public and private medical facilities, including hospitals, clinics, and clinical laboratories in all 32 states, 215,301 of whom correspond to confirmed deaths, and 4,485,163 to recovered patients. Table 1 shows relevant demographic data of COVID-19 patients recorded in the database as of January 31, 2021, breaking down cases by patient condition, gender, age, and state of residence. Note that the data is collected through a form filled by each patient during the admission process at the emergency room, clinic, clinical laboratory, or hospital.

**Table 1:** Demographic characteristics of COVID-19 patients recorded in the database from the Mexican Federal Government, with a cutoff date of January 31, 2021.

Demographic data	Description (Number of cases)
Number of records	(4,700,464)
Patient condition	Deceased (215,301), Recovered (4,485,163)
Gender	Female (2436975) and Male (2263488)
Age group	<18 (214500), 18-40 (2133036), 41-65 (1901409), >65 (451512)
States	1. Ciudad de México (1.4M),
	2. Guanajuato, Estado de México, Nuevo León (500k-200k)
	3. B. California, Coahuila, Jalisco, Puebla, Sonora, Tabasco, San Luis Potosi, Tamahulipas, Veracruz (150k-100k)
	4. Rest of states (90k-20k)

Note that all our algorithms were trained using data obtained from the database with a cutoff date of January 31^th^, 2021. The database includes 28 characteristics for each patient, which can be grouped into three categories: 1) past medical history, 2) demographic data, and 3) information related to the COVID-19 episode. Category 1 (medical history) includes comorbidity information, specifically: diabetes, chronic obstructive pulmonary disease (COPD), asthma, use of immunosuppressive drugs, hypertension, cardiovascular disease, obesity, chronic renal disease, asthma, other chronic illnesses, smoking history, and pregnancy. Category 2 (demographic data) includes gender, age, state of birth, state of residence, whether the patient in question self-identifies as indigenous and/or speaks an indigenous language, is a migrant, or a foreigner. Category 3 (recent medical information) is subdivided into category 3a, which corresponds to those characteristics that may be known by a patient at the point of first becoming ill and receiving medical attention, and category 3b, which corresponds to those characteristics that may become known during the course of medical treatment. Category 3a includes the type of medical facility where the patient is being treated, the state where the medical facility is located, the number of days elapsed from the date of symptom onset to the beginning of treatment, and exposure to confirmed COVID-19 patients. As mentioned, *USMER* is the acronym, in Spanish, used by the General Council of Epidemiology to appoint the health monitoring units of viral respiratory diseases (Unidades de Salud Monitoras de Enfermedad Respiratoria Viral) that integrate the Mexican epidemiological surveillance system. *Sector* refers to the type of institution, belonging to the National Health System, that provided the care. Category 3b includes the COVID-19 status, COVID-19-related pneumonia, hospital and intensive care unit (ICU) admission, as well as the need for mechanical ventilation. Note that the COVID-19 status characteristic defines to which of the seven groups described in the previous paragraph a given patient belongs.

A fourth category of characteristics, which could exhibit a high predictive power as part of our patient classification algorithm, would include information about current symptoms. Such information is unfortunately not currently available in the public database at our disposal. In this sense, our work is a retrospective study which can be improved with the incorporation of additional data in future investigations.

Table 2 summarizes the 28 characteristics and the three categories discussed above. Note that we have displayed in italics 7 characteristics which we have determined to lack a sufficient predictive power and/or yield inconsistent results (as derived from too small a population with the characteristic in question). From this point onward, the characteristics vector employed throughout the manuscript refers to the resulting shortened 21-element vector.

**Table 2:** Classification of characteristics. Characteristics shown in italic font do not exhibit a sufficient predictive power and are therefore not included in our subsequent analysis. We have divided the clinical treatment into four clinical stages to test our neural networks. Stage 1 corresponds to those patients who are in the process of receiving initial medical assessment; therefore, characteristics in categories 1,2, and 3a are assigned. For patients in stage 2, the COVID-19 status and pneumonia characteristics are added to the characteristics vector. When a decision has been made as to whether admit a patient into a hospital or send him/her back home, the hospitalization status characteristic is added at Stage 3. Finally, Stage 4 includes the intubation and ICU characteristics for those patients in critical condition.

Category	Characteristics	
1) Medical History	1. Diabetes	7. Obesity
	2. COPD	8. Other chronic illnesses
	3. Immunosuppressive drugs	*Pregnancy
	4. Hypertension	*Asthma
	5. Chronic renal failure	*Smoking
	6. Cardiovascular diseases	
2) Demographic Data	9. Gender	*Indigeneous
	10. State (birth)	*Indigeneous language
	11. State (residence)	*Migrant
	12. Age	*Foreigner
3) Recent Medical Info	Category 3a	13. USMER designation
		14. Sector (medical facility)
		15. State (treatment)
		16. Days symptoms-treatment
		*Exposure to positive patients
	Category 3b	17. COVID-19 status
		18. COVID-19-related pneumonia
		19. Hospitalization status
		20. Intubation
		21. ICU

### 2.2 Conventional techniques in biostatistics

**Fig 1:**
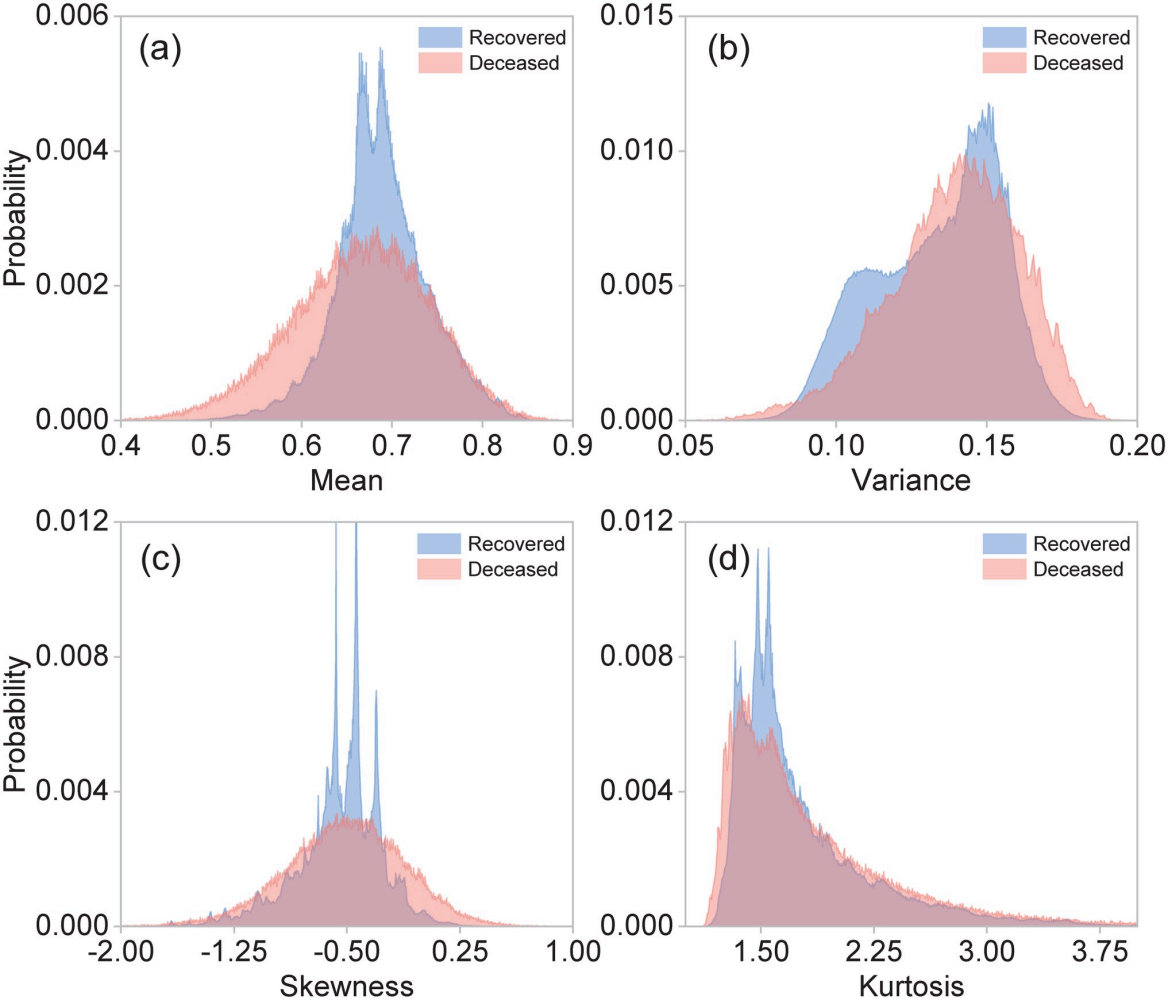
Normalized distributions for different central moments: (a) mean, (b) variance, (c) skewness, and (d) kurtosis, for class 1 (recovered patients), shown in blue and for class 2 (deceased patients), shown in red. These central moments were computed from the 21 elements of the characteristics vector for each patient. These distributions were plotted using the entire dataset with 4, 700, 464 observations.

In biostatistics, Bayesian inference, hypothesis testing, variance analysis, and regression techniques are methods extensively employed for statistical evaluation of medical data [43, 44]. Along these lines, we have directly applied the hypothesis testing method [45] in order to attempt to discriminate between our two classes of patients from the collected data so as to establish a critical bound which may help us to identify the mortality risk for incoming COVID-19 patients. With this goal in mind, we use the first four central moments as estimators. Fig 1 shows the normalized statistical distributions obtained for class 2, i.e. deceased patients (red), and class 1, i.e. recovered patients (blue), using the moments: (a) mean, (b) variance, (c) skewness, and (d) kurtosis. The central moments were computed from the 21 elements in the characteristics vector. Note that the resulting distributions for class 1 / class 2 patients exhibit a large degree of overlap. This implies that the desired classification of patients through the determination of a critical bound is not possible using these estimators.

Bayesian inference constitutes an alternative to frequentist methods, the latter which provide predictions based on the relative frequencies associated with particular events within a large number of trials. Applied to our particular case, Bayesian inference would compute a posterior probability *P*(*C*_*j*_|**x**), where *C*_*j*_ denotes the relevant classes (recovered, *j* = 1, or deceased, *j* = 2), and **x** represents the characteristics vector, resulting from a prior probability *P*(*C*_*j*_) and the so-called likelihood function *P*(**x**|*C*_*j*_) through Bayes theorem [46], *P*(*C*_*j*_|**x**) = *P*(**x**|*C*_*j*_)*P*(*C*_*j*_)/*P*(**x**). Note that in order to determine the likelihood function, *P*(**x**|*C*_*j*_), a model is required in order to estimate the probability of observing a set of characteristics **x** given a known class *C*_*j*_. Although in principle a viable likelihood function could be derived from the sample data, this method tends to fail because the variances in parameter calibration can be rather large along certain directions of the parameter space [6], linked to the large degree of overlap between the distributions for both classes, which has already been discussed. This implies that in our case Bayesian inference does not provide a functional approach for the discrimination between class 1 (recovered) and class 2 (deceased) patients.

The conventional statistical tools and techniques used in clinical medicine are static processes that are consistent and unchanged. In contrast, machine learning dynamically learns and modifies itself as the learning process develops, producing a more robust tool to make predictions. In this context, it has been shown that artificial intelligence methods provide a novel and promising approach for classification and pattern recognition. In what follows, we design, train, and test neural networks in order to identify individual patients as belonging to either class 1 or class 2, relying on historical data, collected from previous patients.

### 2.3 Neural network

Machine learning is a method of data analysis that endows computer algorithms with the capacity to “learn” from a known data-set (which includes defining characteristics and an outcome for each observation), so as to produce a prediction about the outcome given a specific choice of characteristics. Of course, the quality and size of the training data set are crucial in determining the resulting performance of the algorithm. In what follows we demonstrate the use of neural networks trained with the data-set described in Section II. Our neural networks are then used to predict whether a given patient (not included in the data-set used for training) belongs to one of two classes: class 1, which represents those patients who are more likely to survive than to die, and class 2 which represents, conversely, those patients who are more likely to die than to survive.

**Fig 2:**
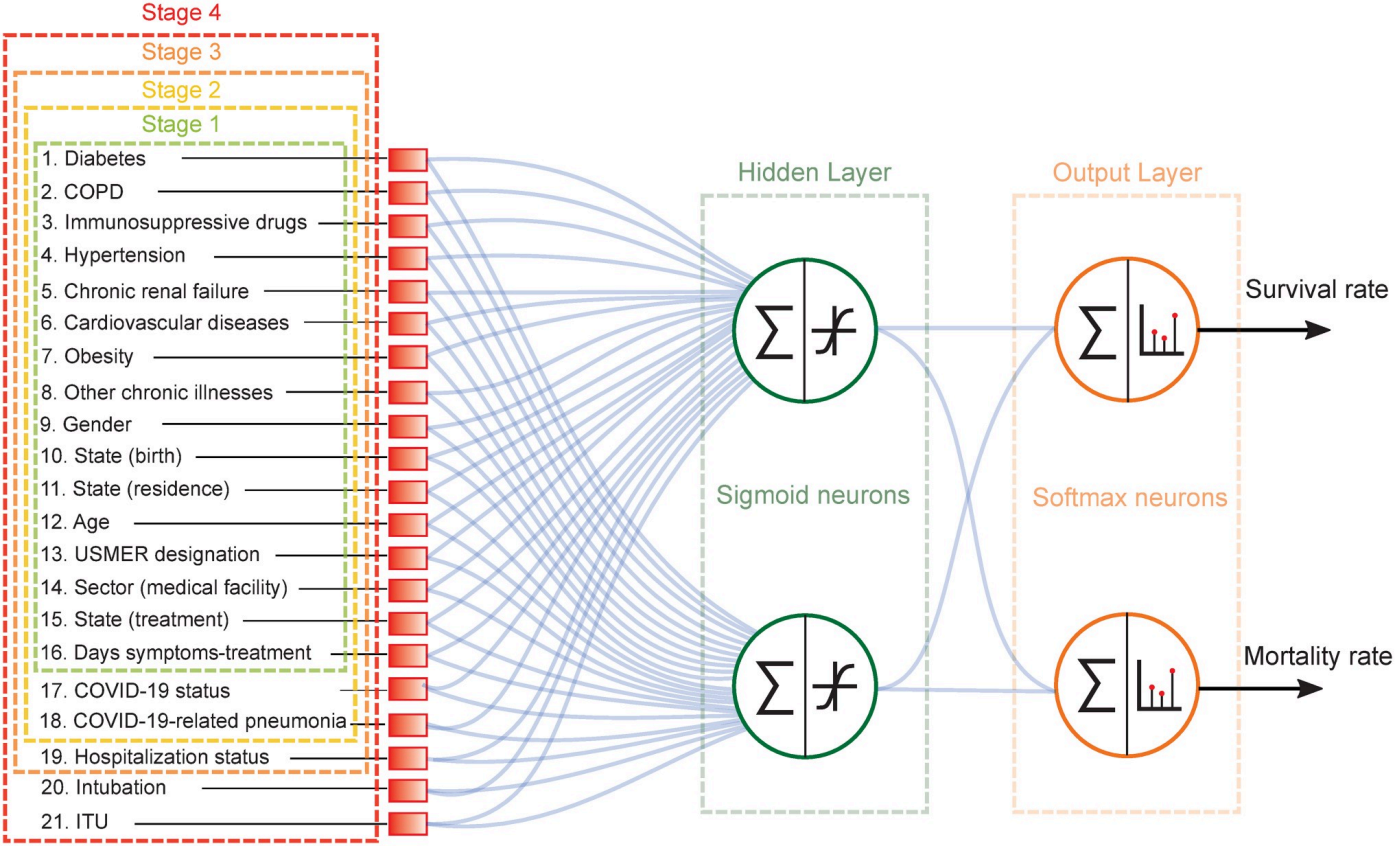
Architecture of the multilayer feed-forward neural network employed in order to classify patients into class 1 (recovered) and class 2 (deceased).

The proposed machine learning algorithm is based on a multilayer feed-forward network with two sigmoid neurons in the single hidden layer and two softmax neurons in the output layer. The hidden layer is indicated by a red, dashed-line rectangle in Fig 2, whereas the output layer is indicated by an orange rectangle. The network’s output represents a probability distribution over the two output classes, which can be interpreted as the survival and mortality probabilities [47, 48]. Fig 2 shows the architecture of the neural networks which we have implemented. The blue lines represent the connections between neurons, each characterized by a synaptic weight. In appendix B we provide a detailed report in which we rank the 21 characteristics by importance as estimated by the synaptic weight magnitude, for each of the two neurons in the hidden layer, and for each of the four clinical stages.

## 3 Results

We have designed and trained four separate neural networks, each corresponding to a distinct clinical stage of the treatment process. Patients in stage 1 are those who are in the process of receiving initial medical assessment and care. For these patients, we can assign data for characteristics in categories 1, 2, and 3a (see Section II and Table 2). Patients in stage 2 are those who as part of their evaluation already have a known COVID-19 status, and may already have contracted COVID-19-related pneumonia. Therefore, the COVID-19 status and pneumonia characteristics are added to the characteristics vector. Patients in stage 3 are those for whom a decision has been reached as to whether admit into a hospital or send back home. Therefore, the hospitalization status is added to the characteristics vector. Patients in stage 4 are those who in addition to being hospitalized, have either been intubated or admitted into an intensive care unit. Therefore, the intubation and ICU characteristics are added to the characteristics vector. Note that in Fig 2, the dashed-colored rectangles indicate the characteristics available in each of the four clinical stages of treatment.

We train our neural networks using the scaled conjugate gradient back-propagation algorithm, while the performance is quantified through the cross-entropy. It is important to point out that, in order to guarantee an unbiased patient classification, we have used a balanced data-set of 430,602 observations, with one half of the observations (215,301) representing all known deceased patients in the database, and the other half (also 215,301) randomly selected from those patients who recovered. We have used a 70%/15%/15% training/testing/validation split of the resulting balanced data-set.

In order to assess the performance of our neural networks, we define the specificity or true negative rate, *TNR*, as the share of true negatives (*TN*)—i.e. true recoveries—to the sum of true negatives and false positives (*FP*). We also define the sensitivity or true positive rate, *TPR*, as the share of true positives (*TP*)—i.e. true deaths—to the sum of true positives and false negatives (*FN*). In addition to the specificity and the sensitivity, we also define the accuracy (*ACC*), as follows

$$TNR=\frac{TN}{TN+FP},$$ (1)

$$TPR=\frac{TP}{TP+FN},$$ (2)

$$ACC=\frac{TP+TN}{TP+TN+FP+FN}.$$ (3)

Once training has been completed, our neural networks can predict with high accuracy whether a given patient with known characteristics belongs in class 1 (more likely to survive), or in class 2 (more likely to die). Across all four clinical stages, our machine learning algorithm exhibits a specificity greater than 82%, a sensitivity greater than 86%, and an accuracy greater than 84%. In general, since more information becomes available for successive clinical stages, it is only natural that the specificity, sensitivity, and accuracy all tend to improve when progressing from one stage to the next. The accuracy, specificity, and sensitivity reach values of 93.5%, 90.9%, and 96.1%, respectively, at stage 4. These three quantities are shown in Table 3 for each of the four clinical stages.

**Table 3:** Accuracy, specificity and sensitivity obtained by the Neural Network (NN), Logistic Regression (LR), Support Vector Machine (SVM) and k-Nearest Neighbors (kNN) algorithms, for each of the four clinical stages.

Stage	Algorithm	Accuracy	Specificity	Sensitivity
1	NN	84.3%	82.4%	86.3%
	LR	82%	83%	83%
	SVM	84%	84%	84%
	kNN	81%	80%	83%
2	NN	90.5%	89.1%	91.9%
	LR	88%	88%	89%
	SVM	88%	88%	89%
	kNN	85%	86%	86%
3	NN	93.1%	90.8%	95.5%
	LR	92%	92%	93%
	SVM	91.8%	91%	93%
	kNN	89.1%	89%	89%
4	NN	93.5%	90.9%	96.1%
	LR	92.1%	92%	92%
	SVM	92.5%	91%	94%
	kNN	89.3%	89%	89%

In order to evaluate the performance of our neural networks, we compare our approach against three different machine learning (ML) algorithms: logistic regression (LR), support vector machine (SVM), and k-nearest neighbors (kNN). LR is a statistical model based on estimating the probability that a given data entry belongs to a certain class. For the binary case, this model uses the logistic function to map the output of a linear equation to the range [0, 1]. If outputs are greater than a predefined threshold value, they are assigned to class 1, otherwise they are classified as class 2 [49]. The SVM algorithm is a machine learning technique that seeks to construct hyperplanes in an N-dimensional space, to separate two sets of data points belonging to different classes. The hyperplanes represent decision boundaries that provide a margin distance so that test data can be attributed to different classes [50]. The kNN method is a supervised machine learning algorithm used for classification and regression problems. This algorithm examines the distribution of the training samples and predicts new cases by calculating a similarity measure, typically distance functions such as the Euclidean distance [51]. Table 3 shows the overall accuracy, specificity, and sensitivity of each predictive model for the four clinical stages. Our results show that our neural networks exhibit a slightly better performance, in terms of accuracy, as compared to the three other ML algorithms that we have considered (LR, SVM, and kNN). In medical diagnosis, *sensitivity* / *specificity* refers to the percentage of correctly identified patients who are affected / unaffected by the medical condition in question. In this context, a higher sensitivity is associated with an enhanced detection rate of real cases, so that sick patients can receive timely medical attention. This accelerates the treatment process, however, at the risk of misallocating resources to patients that do not need such resources. Conversely, a higher specificity leads to the reduction of resource misallocation, but increases the possible omission of real cases [52]. The latter scenario can be dangerous in the context of viruses with a high spreading capability such as SARS-CoV- 2. Remarkably, our neural networks achieve values >90% for both sensitivity and specificity. Our findings show that the LR and kNN methods lead to relatively balanced values for both metrics, whereas NN and SVM present a slight asymmetry with the sensitivity higher than the specificity. This asymmetry suggests that our neural networks favor the efficient identification of high-risk patients during the emergency-department triage process.

Furthermore, note that the simple topology of our algorithm enables low-complexity and low-cost implementations on general-purpose electronic devices that may include mobile phones, tablets, and development boards, which could have important implications for the deployment of this technology in clinics and hospitals [53, 54].

We would like to remark that so as to facilitate the application of our approach, we have developed a practical application that implements the neural networks described in this study. This tool has a user-friendly interface and is publicly available in [55]. This application might facilitate the deployment of this technology across clinics and hospitals, for the real-time identification of high-risk patients. More importantly, it could help identifying the effects of all individual characteristics for each patient in question. Note that the computed survival probability at each of the four clinical stages could serve as a numerical scale to aid in the allocation of medical resources and the management of hospital capacity.

Finally, we would like to stress that while the data at our disposal pertains to Mexico, a similar methodology could be applied to data from other countries, i.e. train and test neural networks using those variables which are available in each particular database of interest. Also note that if we had access to current symptoms data for each patient, the predictions provided by our neural networks would quite possibly exhibit a considerably enhanced accuracy.

### 3.1 Hybrid machine-learning-assisted frequentist hypothesis-testing method

As discussed in Section 3, none of the first four central moments as calculated from the 21 elements of the characteristics vector provide a viable estimator for the hypothesis testing method. In general, the determination of a viable estimator is a challenging task. Here we propose the use of the softmax outputs of our neural networks as estimators, in other words we let the training of our neural networks accomplish the non-trivial task of determining a highly-optimized estimator. This becomes a hybrid technique which exploits both machine learning and standard hypothesis testing.

**Fig 3:**
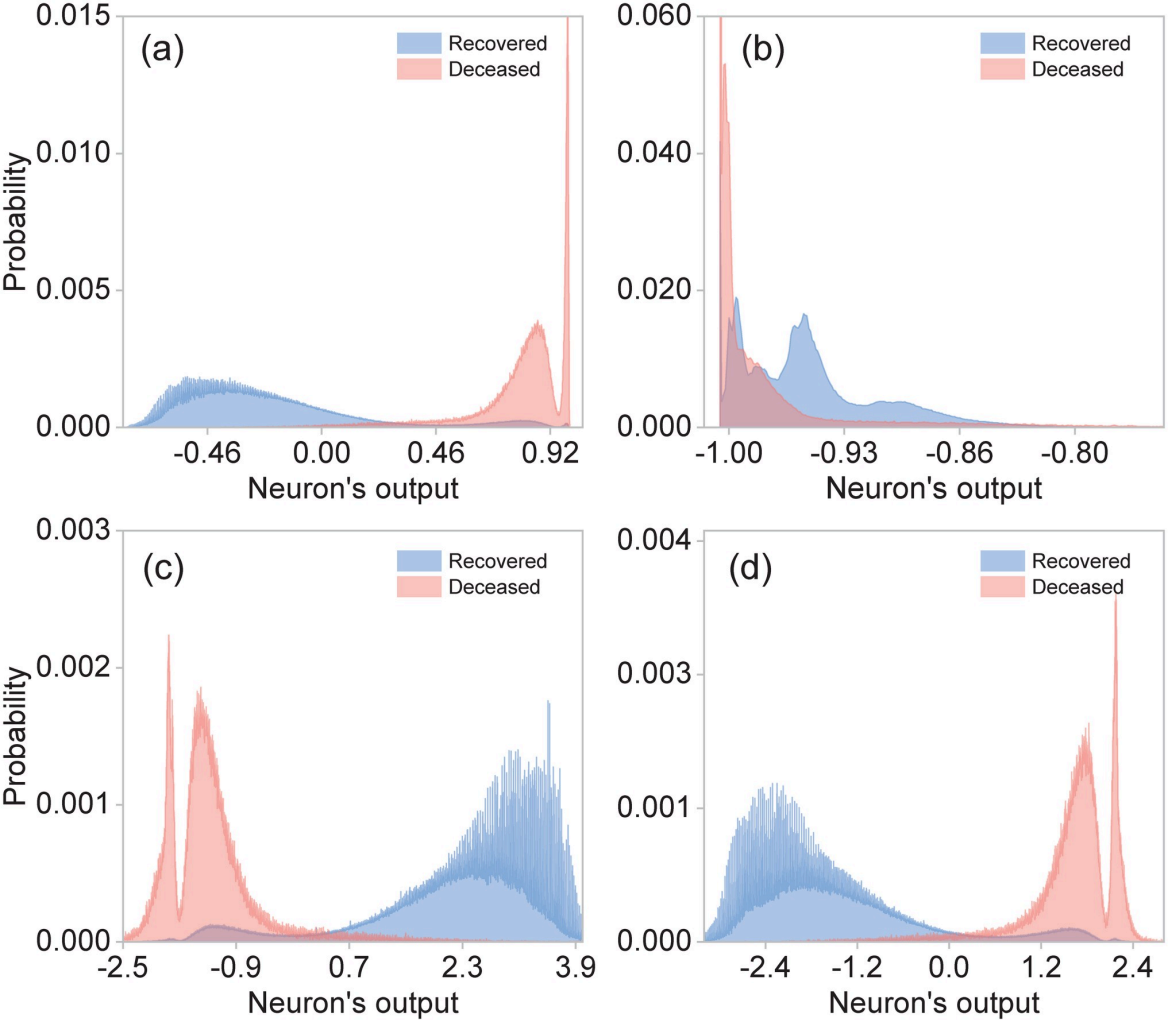
Statistical distributions for deceased (red) and recovered (blue) patients built from the outputs of: (a) neuron-1 in layer 1, (b) neuron-2 in layer 1, (c) neuron-1 in layer 2 and (d) neuron-2 in layer 2.

In Fig 3, we show the resulting distributions of the outputs of each of the four neurons in our neural networks, for those patients known to have died (red), and for those known to have recovered (blue). While the two neurons (particularly the first one) in the internal layer can already do a reasonable job at discriminating between classes 1 and 2, the two neurons in the outer layer accomplish this classification remarkably well.

In order to evaluate the proposed hybrid method, we consider two metrics, Type-I (*α*) and Type-II (*β*) errors, which are defined by

$$pr(\alpha )=pr(Z>p_{\alpha }|H_{0}),$$ (4)

$$pr(\beta )=pr(Z\leq p_{\alpha }|H_{0}).$$ (5)

**Fig 4:**
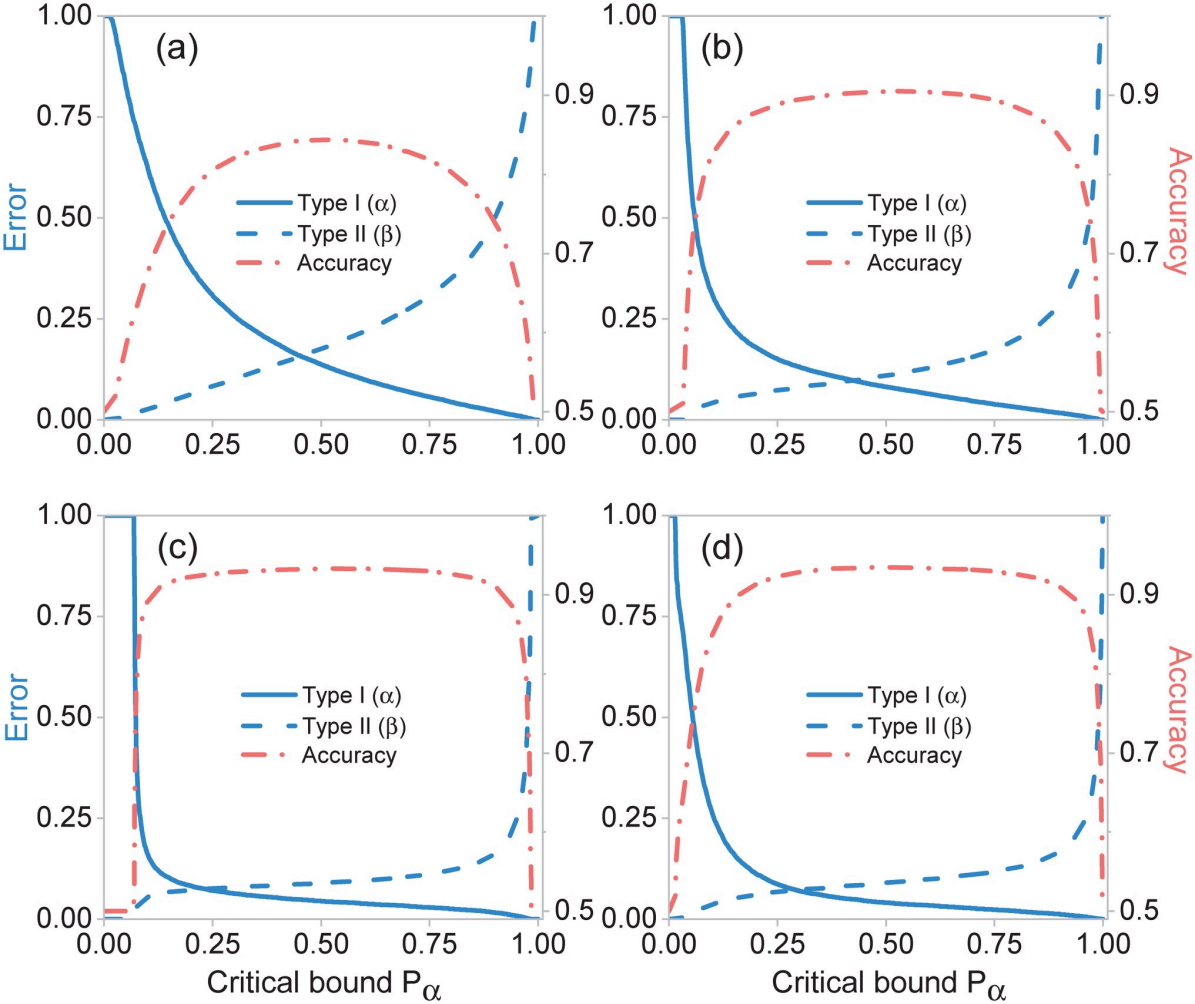
Type-I (*α*) and Type-II (*β*) errors, and accuracies for different clinical stages (a) Stage 1, (b) Stage 2, (c) Stage 3, and (d) Stage 4. Overall accuracy and errors are obtained from the hypothesis testing method as a function of critical bound *p*_*α*_, using the neural network outputs as estimators.

Here, *Z* is the estimator, *p*_*α*_ the critical bound and *H*_0_ the null hypothesis. We set the null hypothesis *H*_0_ to be the death of the patient in question, while the alternative hypothesis *H*_1_ to be the patient’s survival. Fig 4(a) shows the Type-I (*α*) and Type-II (*β*) errors from the hypothesis testing method for the four clinical stages, using the neural network outputs as estimators. Note that the highest accuracies (see Fig 4(b)) of each stage coincide with the accuracies obtained solely with the neural networks (see Table 3).

## 4 Discussion and conclusions

In the midst of this global crisis in which health care systems are overwhelmed, it is of utmost importance to focus efforts on the development of technological tools that allow achieving optimal use of health care resources. In this regard, it is essential to obtain significant knowledge of the prognostic factors associated with COVID-19 for its early identification. In this work, we have presented an effective machine-learning algorithm for the identification of high-risk patients, presenting COVID-19 symptoms. This technology enables rapid identification of high-risk patients at four different clinical stages, ranging from the onset of COVID-19–i.e. at the triage process for patients who arrive at the emergency room–to the need for specialized care including intubation and intensive care units. In order to train our neural networks, we have employed a characteristics vector with 21 elements per patient extracted from a database which includes historical data for 4, 700, 464 confirmed or suspected COVID-19 cases. These 21 elements include information about comorbidities, demographical information, as well as information related to the COVID-19 episode.

We have shown that our neural networks which contain two neurons in the hidden layer are capable of classifying with high accuracy patients into two classes: class 1, comprising those patients who are more likely to survive than to die, and class 2, comprising those patients who are more likely to die than to survive. Furthermore, we have demonstrated that the accuracy, specificity, and sensitivity reach values up to 93.5%, 90.9%, and 96.1%, respectively.

Interestingly, we have shown that the training of our neural networks can accomplish the highly non-trivial task of determining an optimal estimator to be used as part of the standard hypothesis testing method. This results in a hybrid technique that translates the results of our artificial-intelligence-enabled patient classification algorithm into the language of hypothesis testing, commonly used in biostatistics and medicine, thus establishing a bridge between these two disciplines. We believe this result constitutes the foundation for a series of novel strategies for predicting outcomes in clinical medicine, and to enable new perspectives in clinical decision making. It is important to note that our algorithm could straightforwardly run on mobile phones or tablets, which could facilitate its deployment across clinics and hospitals. We point out that our research group plans to carry out prospective studies in which the tool presented here is to be applied to future COVID-19 patients as they seek medical attention, including public and private medical facilities with different budget levels located in urban areas with a range of population levels, thus allowing us to evaluate the ability of this new instrument to make useful predictions. We are certain that our work has important implications in the context of the current pandemic for medical resource allocation and hospital capacity planning.

## 5 Appendix

### 5.1 Hypothesis testing with specific characteristics

**Fig 5:**
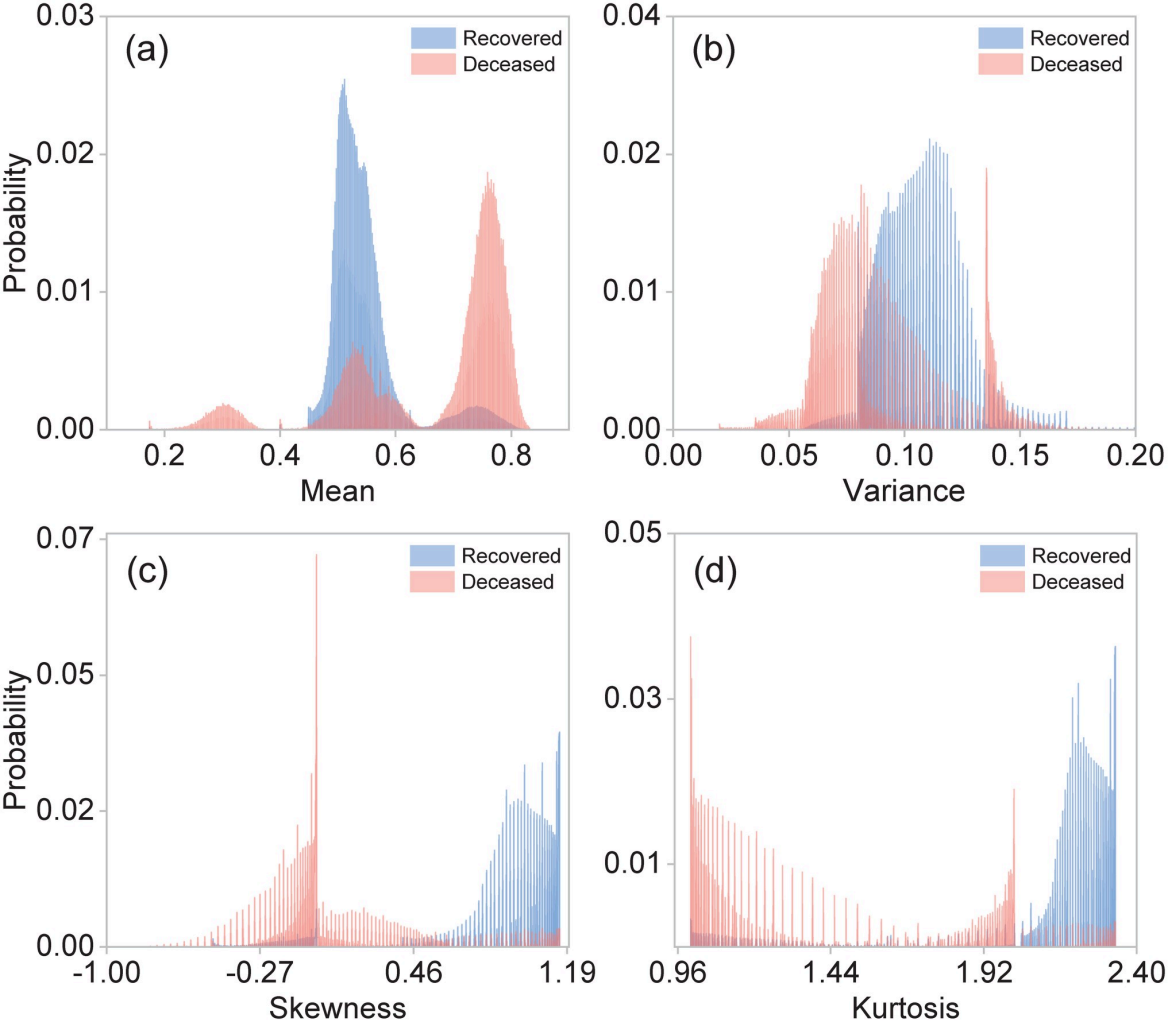
Normalized statistical distributions for different central moments: (a) Mean, (b) Variance, (c) Skewness, and (d) Kurtosis, for class 1 (recovered) shown in blue, and for class 2 (deceased) shown in red. These central moments were computed for a subset of 4 characteristics (age, hospitalization status, intubation and ICU). The distributions were computed from the whole dataset with 4, 700, 464 observations.

As mentioned in Section 3, the central moments computed from the characteristics vector for each patient do not constitute viable estimators to be used in hypothesis testing methods. This is a consequence of the high degree of overlap between the resulting distributions for class 1 (recovered) and class 2 (deceased) patients. With this in mind, we have computed the central moments for a particular subset of characteristics found to be strongly correlated with the outcome (death/survival). Fig 5 shows the resulting distributions for the four first central moments using the following characteristics: age, hospitalization status, intubation, and ICU; class 1 (recovered) is shown in blue while class 2 (deceased) is shown in red. Clearly, the distributions are much less overlapped as compared to the case where all 21 characteristics are used (see Fig 1), allowing us to use these moments as viable estimators for hypothesis testing. Note, however, that the hospitalization status characteristic is only made available at stage 3 of treatment, while intubation and ITU at stage 4. Therefore, unfortunately, these estimators can only apply in an advanced clinical stages where the patients are already in need of specialized care.

### 5.2 Confusion matrices and receiver operating characteristic curves

**Fig 6:**
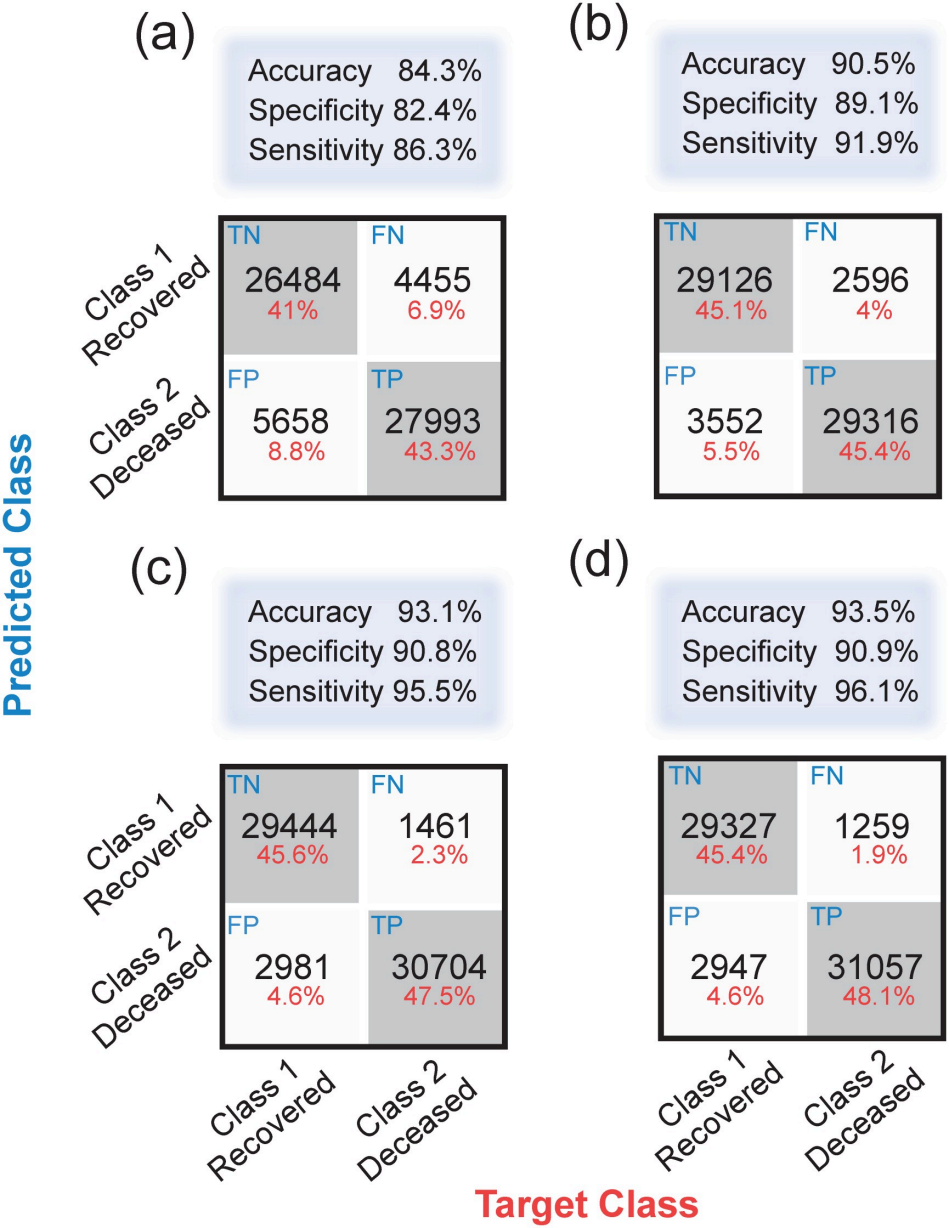
Confusion matrices, which quantify the performance of our neural networks in each of the four clinical stages: (a) Stage 1, (b) Stage 2, (c) Stage 3, and (d) Stage 4. Off-diagonal values correspond to misidentified cases, i.e., false-negatives and false-positives, whereas the diagonal values represent true-positives and true-negatives.

In order to complement the results presented in this work, we provide details about the performance of the artificial neural networks trained for each of the four clinical stages. This is presented in terms of the confusion matrices, accuracies, and receiver operating characteristic curves (ROC). Fig 6 shows the confusion matrices that allow us to assess the performance of neural networks for the rapid identification of high-risk patients in the clinical stages 1 through 4, presented in panels (a) through (d). Note that each matrix is computed from the 15% of observations reserved for testing. While the horizontal axis represents the known outcomes in the test data-set (target class), the vertical axis represents the predicted class using our neural networks (predicted class). Note that diagonal values represent successfully classified patients, i.e. true-positive and true-negatives, whereas off-diagonal elements represent misclassified patients, i.e. false-negatives and false-positives. Fig 6 also displays above each matrix the overall accuracy calculated from Eq (3). Note that in all clinical stages, the accuracy is larger than 84%.

**Fig 7:**
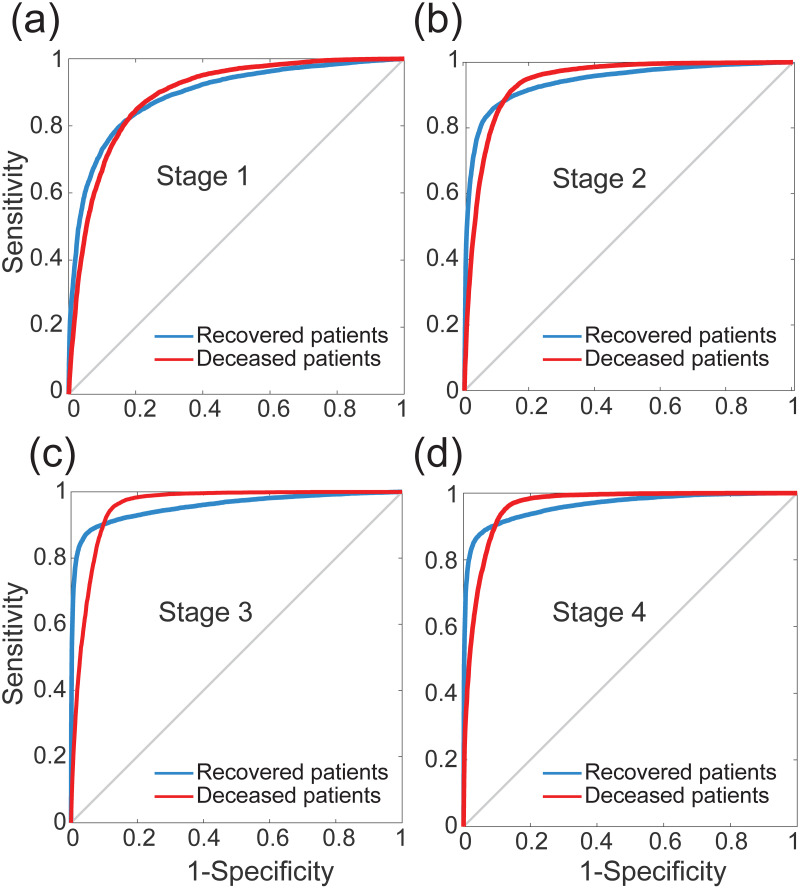
Receiver operating characteristic (ROC) curves that illustrate the diagnostic ability of the neural networks for rapid identification of high-risk patients in each stage: (a) Stage 1, (b) Stage 2, (c) Stage 3, and (d) Stage 4. The blue lines correspond to recovered patients, whereas the red lines depict the deceased patients.

Fig 7 shows the ROC curves of our neural networks for rapid identification of high-risk patients in each of the four clinical stages 1 through 4, shown in panels (a) through (d). These curves represent the sensitivity and specificity values computed across all possible threshold values. Here, the sensitivity is inversely related to the specificity, i.e., as sensitivity increases, the specificity decreases. Note that the curves shown in Fig 7 provide equivalent information to the one presented in Section 3, namely hypothesis testing methods in terms of Type-I and Type-II errors.

**Fig 8:**
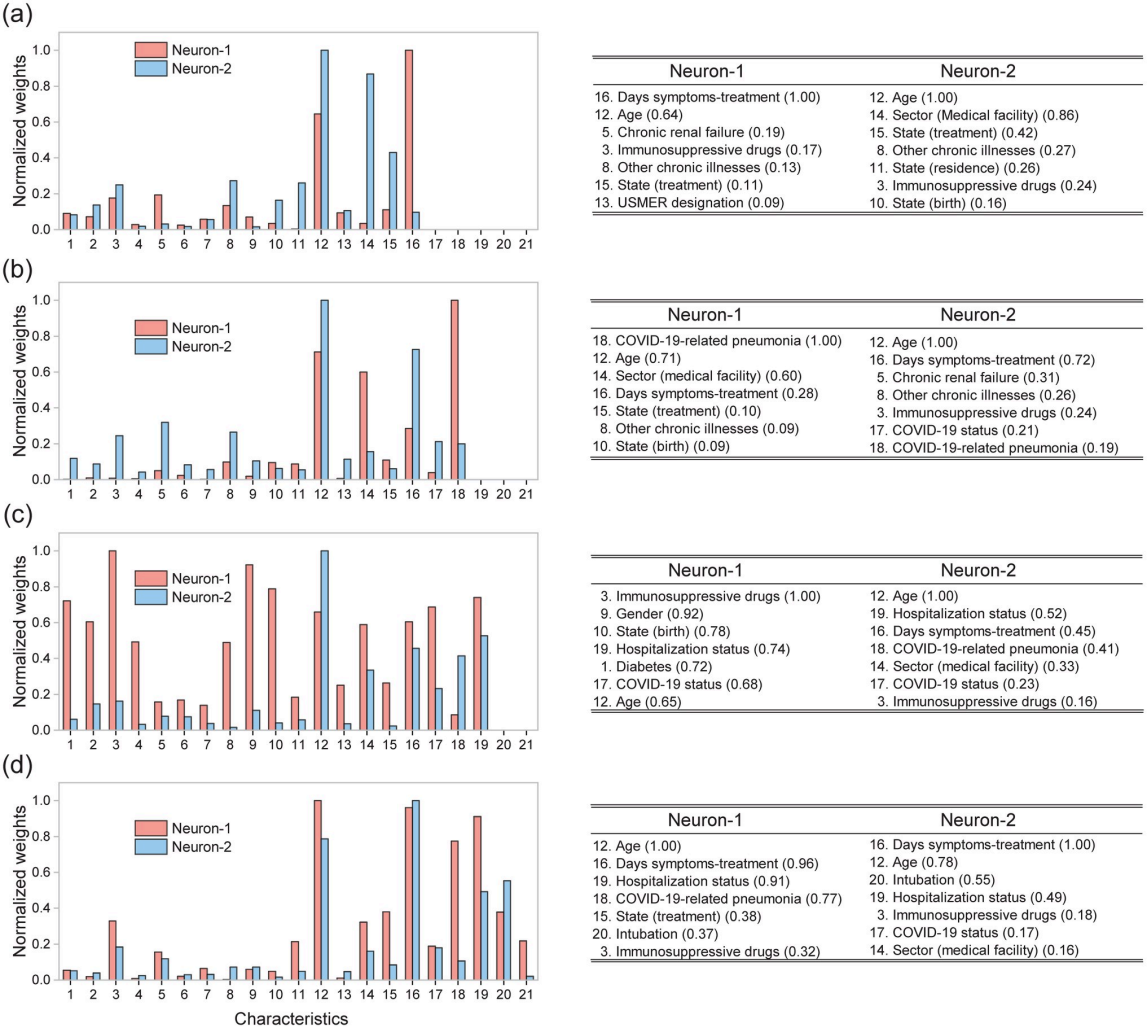
Left: Normalized absolute values of the synaptic weights for the two sigmoid neurons in the hidden layer for each clinical stage: (a) Stage 1, (b) Stage 2, (c) Stage 3, and (d) Stage 4. Right: tables describing the seven dominant characteristics for each of the two neurons the hidden layer, ranked by the absolute value of the synaptic weight (value shown within brackets).

Note that from our analysis it becomes possible to compare quantitatively the relative importance of each of the 21 characteristics in defining an outcome (death/survival) for a given patient. In Fig 8 we plot the normalized, absolute value of the synaptic weights for neuron 1 (shown in blue) and neuron 2 (shown in red) as a function of the characteristic number (1 through 21, see Table 2), for each of the four clinical stages 1 through 4, shown in panels (a) through (d). We have also included from each of the two internal-layer neurons and for each of the four clinical stages, a list of the seven dominant characteristics, ranked by the absolute value of the synaptic weight (value shown within brackets). Interestingly, we find that the age, number of days elapsed from the date of symptom onset to the beginning of treatment, and use of immunosuppressive drugs are variables with a significant influence in almost all clinical stages, for both neurons in the hidden layer. Note that state (treatment) is an important characteristic in three out of four treatment stages for neuron 1, while sector (medical facility) and COVID status are important for neuron 2. Some characteristics such as other chronic illnesses, state (birth), COVID-19-related pneumonia, and hospitalization status impact moderately on the final decision. Note that some comorbidities including diabetes, hypertension, cardiovascular diseases, and obesity do not appear amongst the seven dominant characteristics at any of the clinical stages.

### 5.3 Estimation capacity of neural networks

**Fig 9:**
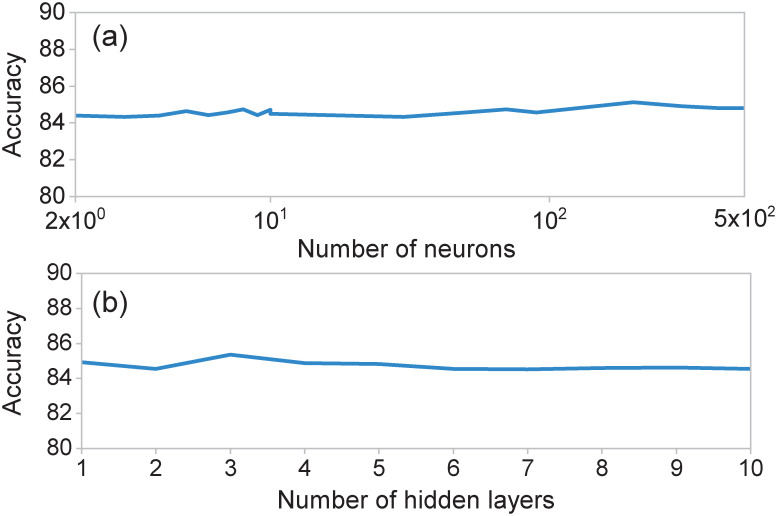
Overall accuracy of the neural network for the identification of high-risk COVID-19 patients, at the first clinical stage, as a function of (a) the number of neurons in the hidden layer and (b) the number of hidden layers. In the last case, the number of neurons in each layer was set to ten.

In order to determine the optimal topology for our neural networks, we monitored the resulting performance of the trained neural network as a function of the number of layers and number of neurons per layer. As an illustration, we preset here results for neural networks related to the first clinical stage; note, however, that we have obtained similar conclusions for the neural networks in the remaining clinical stages. While Fig 9(a) shows the overall accuracy as a function of the number of neurons of a single-hidden-layer network, Fig 9(b) shows the overall accuracy vs the number of layers, fixing the number of neurons per layer to ten. As may be appreciated form the figures, the accuracy exhibits essentially a flat dependence on number of neurons and on number of hidden layers, justifying our neural network design involving a single, two-neuron hidden layer. Importantly, an architecture based on a hidden layer with two neurons enables us to save computation time and implement the algorithm easily.
